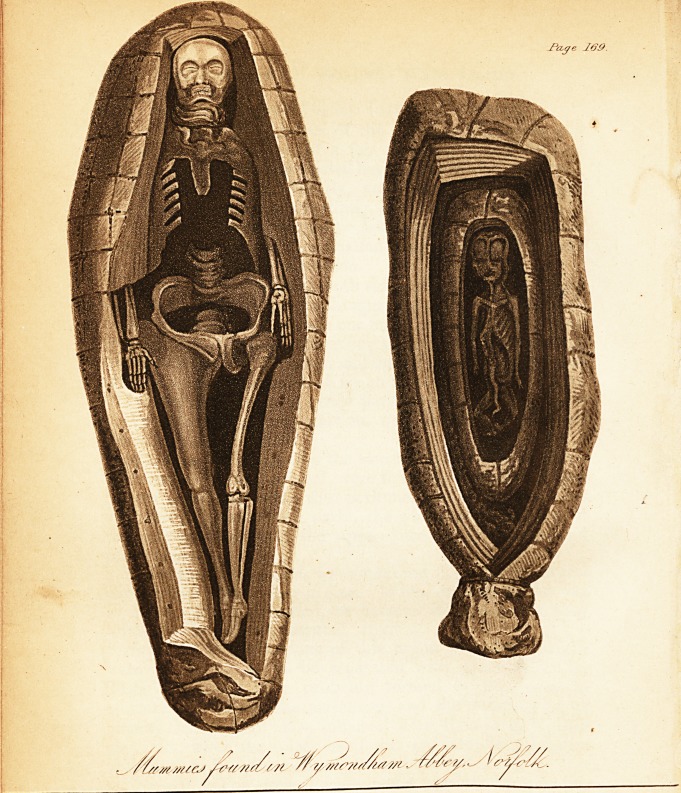# An Account of the Examination of Two Bodies, Found in the Vaults of the Ruins of Wymondham Abbey, in Norfolk

**Published:** 1834-10-01

**Authors:** John Dalrymple

**Affiliations:** Assistant Surgeon to the London Ophthalmic Infirmary.


*An Account of the Examination of two Bodies, found in the Vaults of the Ruins of Wymondham Abbey, in Norfolk.* By John Dalrymple, Esq., Assistant Surgeon to the London Ophthalmic Infirmary.

[with an engraving.]

On the 23d of December, 1833, whilst some labourers were engaged in clearing away a portion of the ruins of the old abbey of Wymondham, a market-town in the neighbourhood of Norwich, a vault was discovered, and laid open, containing two leaden coffins, of very different dimensions. From the great antiquity of the ruins, these relics excited much curiosity among the inhabitants of the place; and the larger of the two coffins was without loss of time divided throughout, in the direction of its length. Within was found not the mouldering fragments of a skeleton, but an entire human body, carefully wrapped in cerecloths, and to all appearance in a state of high preservation. At this point curiosity for the present stopped; further investigation was deferred, and the coffins, for safety, conveyed to the interior of the adjoining church, until a more minute and satisfactory examination could take place.

On the fifth day after the discovery thus made by the workmen, my father was requested to go over from Norwich, to make an inspection of the body contained in the larger coffin; but, being prevented by unavoidable circumstances from accepting the invitation, I took his place, and, accompanied by one of my brothers and by Mr. Woodward, an excellent antiquarian, residing in Norwich, I proceeded to Wymondham, and, in the presence of the clergymen, and several medical and other gentlemen of the place, began my examination of the newly discovered body.

The two coffins were of very unequal dimensions: the larger measured six feet two inches in length, thirteen in breadth at its widest part, and ten inches in depth; it tapered slightly from head to foot. The small one, still unopened, measured only sixteen inches and a quarter in length, by six inches in breadth at the head, where it was five inches and a half deep. This one was also somewhat narrower at the feet than at the opposite extremity, and four inches in depth. The lead of both coffins was very fresh in its appearance, and had lost so little of its metallic lustre, that its line of solder was still bright. Neither coffin bore any date or inscription, by which its antiquity, or the name of the body it contained, could be ascertained. There were no traces of any wooden casing exteriorly, and I believe I

am correct in stating there were none within. Owing to some fragments of the brick vault having formerly fallen in, a portion of the lead, at about the centre of the left side of the coffin, was crushed, and admission given to both air and moisture. It may be recollected that an accident of nearly the same kind, and followed by similar results, had happened to the coffin of Henry the Eighth, as mentioned in Sir Henry Halford's admirable paper on the discovery of the remains of King Charles the First.

The length of the larger coffin internally measured five feet eleven inches; the extreme length of the body in its wrappers was five feet nine inches.

The general form of the body was not unlike that of an Egyptian mummy. The linen bandages were, however, *throughout* saturated with a resinous compound, smelling strongly of gum galbanum, and burning with a bright flame; the whole was carefully and neatly bound, both longitudinally and transversely, by a cordage rather thicker than a swan's quill. Although this cordage was nowhere actually disjoined, and the knots were still entire, yet it was so softened by the influence of moisture, that the least touch was sufficient to break up its texture. The anterior stratum of the investments of the body was of a light brown colour, somewhat decomposed, and easily removed in flakes. The impressions or marks of the threads of the linen remained, but the fibres had generally disappeared, and its consistence seemed owing to the resinous nature of the composition in which it had been immersed, when liquified by heat.

In many places we found the wing-cases and other remains of a small species of beetle lying upon the compound, and towards the extremity of the body the composition was much honey-combed by the labours of these insects. Some living larvæ found at the examination were taken, in order to witness their transformation into the perfect insect.

Upon comparison, these minute Coleoptera appear to be identified with the Cerylon Hysteioides of Latreille, which live under the bark of trees. The larvæ also probably subsisted upon the vegetable fibres of the linen or fragments of wood mixed with the resinous composition of the outer stratum.

Beneath the exterior surface, fresh wrappers of coarse cloth succeeded, again and again bonded with cordage, stronger and more perfect as we approached the body; and at either extremity of the body so firm were the investments, and so thoroughly saturated with resins, that it required a strong knife to remove them sufficiently to expose the enclosed body. The cords which bound the outer and inner strata of linen rolls were applied with great nicety and regularity, and, by intersecting each other at right angles, where they were firmly knotted, the surface was chequered by squares of equal size. On reaching the surface of the body, the sternum and ribs first presented themselves, black, and bare of cartilage. The thorax and abdomen were filled with a dark-coloured pultaceous mass, among which no trace of any viscus was perceptible.

The left arm and hand were without flesh, but the size of the bones at once determined the question of sex; they must have belonged to a female of small stature. The pelvis was ample, the distance measured from the anterior and superior spinous processes of the ilia giving nearly nine inches; from the promontory of the sacrum to the pubes, five inches; and the outlet (antero-posterior diameter), four and a quarter inches. Of the left thigh and leg nothing remained but their bones; while on the right side the skin and muscle still existed. The length of the thigh-bone was sixteen inches, of the leg fourteen and a half; of the foot from heel to toe seven and a half inches. In respect to the position of the feet, they were placed somewhat across each other, and were extended into nearly a straight line with the body.

The viscera of the pelvis were still distinct; the organs of generation had been stuffed with flax mixed with spices. The bladder was collapsed. The rest of the pelvic viscera were partly converted into adipocire, mingled however with distinct brownish fibres, the remains of the iliac and psoas muscles. The hair still adhered to the skin of the pubes.

On exposing the head of the body, a large mass of long curling hair was found, placed round the neck and under the chin, of a glossy brilliancy and auburn colour, approaching to a reddish tinge: it had evidently been shaved from the head, and carefully arranged in the above position.

From the tenacity of the cerecloths at this part, I had hoped to have been enabled to have removed them as a mask, and so have preserved a mould of the features of the individual person: this, however, I found to be impracticable, the integuments being still adherent. The eyeballs had not been removed from the orbit, but, though shrivelled and retracted, (the eyelids falling in upon them,) were still distinct. The nostrils were stuffed with flax; the lips had disappeared, exposing a beautiful set of small and well-arranged teeth, the cutting edges of which had been but little worn. The scalp separated easily, and with that peculiar crackling noise which attends this operation in the recent subject: the cellular tissue was, in fact, perfect. The ears, though wanting, had left their impressions in the waxen cloths, showing that the bandages were applied while the resinous compound was yet liquid. The total length of the body did not exceed five feet.

Some surprise was excited among the medical practitioners who were present, on finding that the capacity of the uterus equalled that of a small orange. This circumstance was explained by the contents of the lesser coffin, which we now proceeded to examine.

Within this second leaden case we found a small mummy, about thirteen inches long, hard, dry, and firmly bound with cords, tied at one end like the neck of a sac. The cerecloth of which it was composed was so fully impregnated with resin, which had been melted over it, that it was found necessary to use a saw to open its cavity. In dividing to about one and a half inch, a hollow was exposed, containing a second package surrounded by a bed of cummin-seeds, so fresh and dry as to yield a very powerful odour. This interior case was carefully corded like the first, and when opened showed a cavity filled with cummin and coriander seeds, nitre, common salt, and fragments of fragrant woods, producing a rich and pleasant perfume. In the centre of this bed was deposited a fœtus, about four and a half inches in length, black, dry, and flattened; the umbilical cord still remained; the sex unascertainable. This at once explained the circumstance of the uterus having admitted of being stuffed, and it led us to infer the probability of the female having died pregnant, and that, on embalming the body, the uterus had been opened, and the fœtus removed. This, I think, is more probable than that she had died in consequence of a miscarriage, as in that case the uterus would have been more contracted than we found it to be. As I before observed, there was no mark or date upon the coffin; neither was there any ring or ornament upon the body, or in the coffin. It was evident, however, from the care which had been taken in the preservation of the corpse of the mother, as well as of the fœtus, that it was a person of high rank, most undoubtedly young, and possibly dying pregnant of her first child.

The ruins of the abbey among which these curious remains were found are of considerable antiquity. Mr. Woodward, by measurement, ascertained that the vault was placed in the central line of the chancel, in the situation of the high altar; a position appropriated to the burial of the more illustrious personages. Old documents also prove that the founder erected this structure about the year 1120, and buried his wife before the altar, she dying in early life.

The abbey of Wyndham, or Wymondham, was founded in the year 1120, by William de Albini, or Daubeny, who married a daughter of Ralph Bigod, Earl of Norfolk.

For the gratification of the collectors of legendary tales, I may mention, that a clergyman residing in the town informed me he had often heard a report among the lower classes, of a golden cradle being somewhere buried among these ruins. In almost every place where the remains of old castles, monasteries, &c. are to be found, there have existed, time out of mind, a race of *treasure-hunters*, and it is not improbable that the story of this interment had been handed down from the earliest period to the present time. The leaden coffin of the fœtus has been gilded by the imaginations of the romantic, and its narrow cell converted into the cradle of the infant of a once illustrious but extinct family.

Since the above examination was made, further excavations have brought to light some stone coffins, in which were found the skeletons, more or less perfect, of other persons who had been buried in the abbey, and which, from the remains of hair-cloth and other vestments, appear to have belonged to the common order of Benedictine monks. No traces of any process of embalming were, however, discovered. I have also ascertained that, about twenty years ago, the vault in which the coffins were found had been accidentally opened; but foul air having extinguished a candle, let down into the cavity by way of precaution, no person ventured to explore its contents. The vault was therefore hastily closed up, and all recollection of the circumstance gradually died away. It seems exceedingly probable, therefore, that the fracture in the side of the larger coffin was produced by the bricks and rubbish falling in through the carelessness of the workmen employed; and that, but for this accident, the body of the mother would have equalled in its preservation the best specimens of the Egyptian art.

8, *New Broad street;*
*August* 8, 1834.

## Figures and Tables

**Figure f1:**